# Low-Latency Haptic Open Glove for Immersive Virtual Reality Interaction

**DOI:** 10.3390/s21113682

**Published:** 2021-05-25

**Authors:** Donghyun Sim, Yoonchul Baek, Minjeong Cho, Sunghoon Park, A. S. M. Sharifuzzaman Sagar, Hyung Seok Kim

**Affiliations:** 1 Department of Intelligent Mechatronics Engineering, Sejong University, Seoul 05006, Korea; simdh2073@sju.ac.kr (D.S.); wmolnaf@sejong.ac.kr (Y.B.); s.park@sejong.ac.kr (S.P.); 2 Department of Information and Communication Engineering, Sejong University, Seoul 05006, Korea; 17013130@sju.ac.kr

**Keywords:** immersive VR interaction, haptic open glove, rotary position sensor, MMS filter, hand motion capture, human computer interaction

## Abstract

Recent advancements in telecommunications and the tactile Internet have paved the way for studying human senses through haptic technology. Haptic technology enables tactile sensations and control using virtual reality (VR) over a network. Researchers are developing various haptic devices to allow for real-time tactile sensation, which can be used in various industries, telesurgery, and other mission-critical operations. One of the main criteria of such devices is extremely low latency, as low as 1 ms. Although researchers are attempting to develop haptic devices with low latency, there remains a need to improve latency and robustness to hand sizes. In this paper, a low-latency haptic open glove (LLHOG) based on a rotary position sensor and min-max scaling (MMS) filter is proposed to realize immersive VR interaction. The proposed device detects finger flexion/extension and adduction/abduction motions using two position sensors located in the metacarpophalangeal (MCP) joint. The sensor data are processed using an MMS filter to enable low latency and ensure high accuracy. Moreover, the MMS filter is used to process object handling control data to enable hand motion-tracking. Its performance is evaluated in terms of accuracy, latency, and robustness to finger length variations. We achieved a very low processing delay of 145.37 μs per finger and overall hand motion-tracking latency of 4 ms. Moreover, we tested the proposed glove with 10 subjects and achieved an average mean absolute error (MAE) of $3.091^{\circ }$ for flexion/extension, and $2.068^{\circ }$ for adduction/abduction. The proposed method is therefore superior to the existing methods in terms of the above factors for immersive VR interaction.

## 1. Introduction

### 1.1. Motivation and Challenges Definition

In the past decade, the influence of tactile and haptic technologies has increased significantly. Haptic technologies enable users to sense, control and manipulate objects through a virtual reality environment. Moreover, they provide tactile or haptic feedback to users through tactile actuators. Compared to other available technologies at present, tactile Internet remains in the innovation phase. One of the enabler technologies of the tactile Internet is haptic communication. However, with haptic communication, extremely low latency, high reliability, and adaptability are required to provide a smooth virtual reality experience. Researchers have determined that to enable a smooth haptic communication experience, an end-to-end latency of 1 ms is required in some scenarios [1,2,3,4]. Numerous haptic gloves have been developed to deliver haptic sensation and allow for controlling and manipulating objects in real time over a network. However, these gloves do not consider the delay caused by data processing, which adds to the latency in the haptic communication loop. Moreover, most of these gloves are not open-hand type, which degrades their robustness and adaptability because the gloves can be worn by users with different hand sizes and hand lengths. The present study addresses these challenges by proposing a low-latency haptic open glove. The proposed glove uses two small rotary position sensors placed in the metacarpophalangeal (MCP) joints to detect finger flexion/extension and adduction/abduction. The acquired data are then processed using min-max scaling (MMS) filter to provide tactile experiences through virtual reality.

### 1.2. Related Work and State of the Art

Immersion can be defined as the perception of being physically present in a virtual-world domain (VWD), where users can fully interact with different objects. The content expressed through the transmission of the information that causes users to become immersed is called immersive content [5]. In recent years, immersive content has been used in various applications; typically, it is used in extended reality applications, including virtual reality (VR) and mixed reality (MR) [6,7]. Notably, immersive content provides realism because next-generation media closely resemble the real-world domain (RWD). Many VR technologies use head-mounted displays (HMDs) to provide RWD interaction in the VWD. HMDs actively provide VR content as immersive content and have evolved to provide immersion beyond the virtual domain [8]. However, these interface devices must minimize the gap between the target content and the RWD reproduced in the HMD to provide a smooth immersion experience. VR interface devices connect the VWD and the RWD, and users can use natural user interfaces (NUI) such as voice, touch, motion, and even brain waves to operate these devices [9]. Researchers are exploring different technologies such as Leap Motion, and Ultra-Leap Motion to provide virtual immersion [10,11]. Masurovsky et al. and Luimula et al. explore the possibility of controller-free virtual immersion using Leap Motion technology [12,13]. Alakhawand et al. introduced a biomimetic tactile fingertip to measure haptic stimuli in mid-air [14]. Jorge Cardoso presents Leap Motion-based VR locomotion techniques as to alternative to gamepad and gaze detection-based locomotion techniques [15]. However, the proposed techniques require more effort than the gamepad controller. Moreover, the performance of the Leap Motion-based VR locomotion technique is not up to the level of the gamepad. Nevertheless, the proposed technique is preferable for some activities. Although the Leap Motion technology is promising, it has significant latency in tracking the human hand. This has been proved by Silva et al. [16]. Leap Motion controllers also work on a line-of-sight theory. It cannot detect hand gestures when users move their hands out of range, which affects these devices’ robustness for a wide area of application. It also cannot distinguish between different fingers when the fingers are very close to each other and vulnerable to occlusion [10,17]. Ultra-Leap ultrasound devices also have vulnerabilities such as noise, physiological limitations, precision, and safety issues. These devices have a very short range of focal points which affects the spatial resolution of these devices [18]. Moreover, the use of surgical gloves during telesurgery or teleoperation may cause the precision of the ultrasound haptic devices [19]. Moreover, long time exposure to the ultrasound wave may cause different medical conditions such as hearing loss [20,21]. On the other hand, haptic gloves do not have these shortcomings as most of the haptic gloves use sensors attached with hand to track angular movements of the fingers’ different joints. Moreover, life-critical applications such as telesurgery, telemedicine, teleoperation need very precise haptic and tactile feedback. To provide precise motion-tracking and haptic-tactile feedback, gloves-type devices are introduced as different actuators, and sensors can be embedded in these gloves. Gloves capable of motion detection have been used in various VR applications [22,23]. For example, games or training materials for improving drivers’ driving skills are provided through HMDs, where gloves are used to represent hand movement in the VWD to create RWD immersion. However, to provide RWD immersion, it is necessary to deliver to users virtual sensations from the VWD to the RWD.

Several haptic gloves and devices have been introduced over the years to deliver virtual sensations. Perret et al. classify different types of haptic gloves present in the commercial market based on traditional, thimbles and exoskeleton gloves [24]. The authors also presented specifications of those haptic gloves in force-feedback, tactile feedback, motion-tracking, etc. One of the early attempts to enable virtual sensations in the RWD includes the development of PHANTOM [25]. This device uses a user’s hand position through a robotic arm and provides stiffness by employing three DC brushed motors. Senso introduced a vibration-motor-based glove to interact with fingers through a vibration motor placed in each finger [26]. This device uses IMU sensors to measure finger movements in space. It achieved a latency of 15 ms in virtual interaction. ContactCi introduced exotendons and servomotor-based gloves that used flex sensors and vibration cues to provide haptic interaction [27]. The resulting haptic interface yielded a latency of less than 10 ms. One of the most well-known haptic gloves, called CyberGrasp, integrates conventional dataglove and exoskeleton mechanisms to detect hand movements and implement cable-based force-feedback systems [28]. Cybergrasp uses Ethernet as a communication method to reduce latency. Dexta robotics introduced exoskeleton force-feedback gloves using a rotary sensor for each finger to measure finger movement in terms of abduction and flexion [29]. This device also uses a servo motor in its force-feedback mechanism, and its response time is 50 ms, including processing and control time delay. Haptx provides precise haptic feedback through 100 pneumatic tactile actuators [30]. This device uses magnetic sensors to capture sub-millimeter finger movements, allowing for the acquisition of highly detailed movement information and the provision of precise force-feedback. Sense glove, introduced by a Dutch company called SENSEGLOVE, uses IMU sensors to track hand motion and provide 23 degrees of freedom (DoF) [31]. Moreover, it provides both vibrotactile and kinesthetic haptic feedback interaction. Its vibrotactile feedback uses six vibration actuators for each fingertip, and its force-feedback mechanism uses a servo motor for each finger. The overall haptic feedback response time is less than 10 ms, including the time required for processing and implementing force-feedback delay.

Researchers have pointed out that an end-to-end latency of 1 ms is required to enable tactile Internet for human-machine interaction in a teleoperation closed-loop system and for providing various VR-based contents. Xiang et al. pointed out that roughly 0.1 ms can be allocated to haptic devices for processing purposes to meet the rigorous requirements of the tactile Internet [1]. Although researchers are attempting to introduce robust and feasible haptic devices, latency reduction remains an underexplored field. Based on a review of the above studies, it seems imperative to introduce haptic devices with extremely low processing delay.

Most of the traditional gloves are garment-made flexible closed-type gloves [24]. In closed-type gloves, sensors and actuators for feedback system are sewn or fixed outside the glove. These gloves also have some drawbacks. Sensors and actuators used in these devices need to be very small to fit under the garments. Moreover, the glove system needs to be flexible and adjustable, or users can feel uncomfortable manipulating objects. These devices also need to be robust to accommodate deformation because users with different hand shapes can frequently use them. One of the main disadvantages of closed-type devices is usability issues. People’s finger sizes are not the same; people with bigger or smaller hand sizes than the actual gloves can feel uncomfortable wearing gloves. Moreover, sensors and actuators are fixed in the specific place in the glove, and to be able to achieve full immersion user’s hand needs to be fitted with gloves length. Furthermore, long time use of closed-type gloves can also cause irritation and sweating problems. As most of the closed-type gloves are made of garments, they can become dirty over time which causes hygienic problems. Lastly, users with any contagious virus can be spread through gloves because many users can wear gloves, and it is very difficult to clean the gloves due to their structure. Although our brains can take visual presentation as the dominant information, some life-critical applications such as telesurgery, teleoperation, telemedicine need a very high level of sensation, high motion-tracking accuracy, low latency, haptic, and tactile feedback as well. Considering these factors, we have proposed an open-type glove to achieve low processing delays with high motion-tracking accuracy.

**Figure 1:**
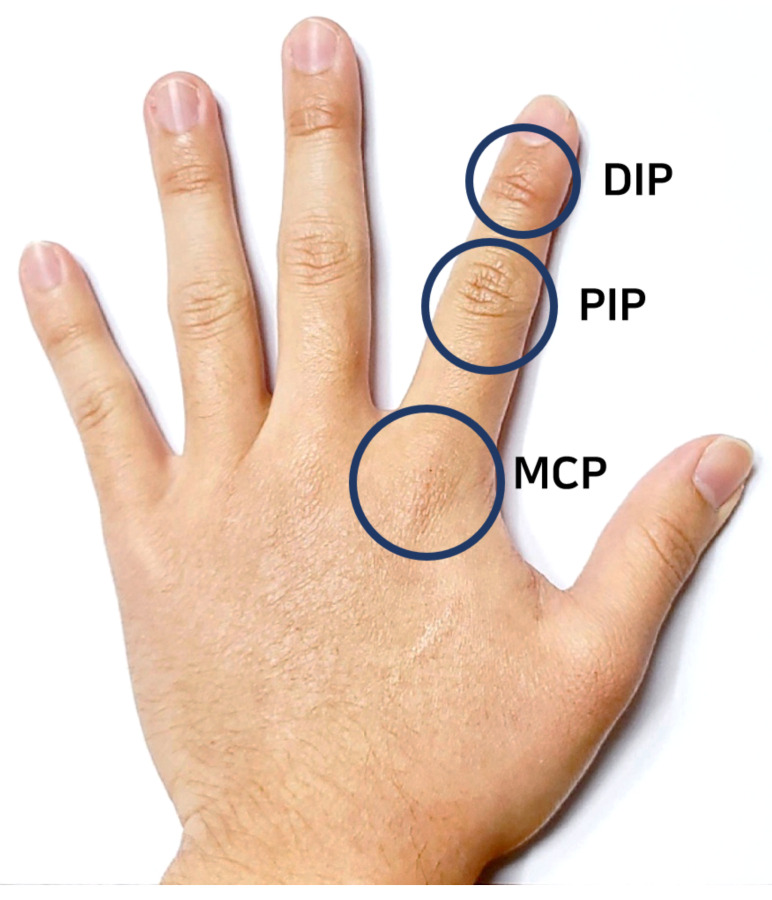
The basic hand anatomy of humans consists of MCP, PIP, and DIP joints.

Figure 1 shows the basic hand anatomy of metacarpophalangeal (MCP), distal interphalangeal (DIP), and proximal interphalangeal (PIP) joints. MCP is the joint at the base of the finger which connects the palm with the basic structure of the finger. Normal flexion-extension can be measured by calculating the displacement of MCP joints. PIP is the joint in the middle of the finger. It is also responsible for flexion-extension for more complicated hand motion. DIP is the joint closest to the fingertip. The displacement of this joint during flexion-extension is not as close to other joints. Different techniques are being used in the literature to track hand motion by measuring the displacement of MCP, DIP, and PIP joints. The technique presented in [32,33] detects motion by sensing the degree of flexion and extension at DIP and PIP joints of the fingers using the flex sensors. However, due to the finger’s structural restrictions, accurate motion-tracking can only be possible if two flex sensors are wrapped with the finger. In this scenario, one flex sensor will track DIP joints movement, and another flex sensor will track PIP joints movement. The motion detection method presented in [34,35] collects necessary data using an inertial measurement unit (IMU) sensor. One of the advantages of IMU sensors is that they can be used in open- and close-type gloves, whereas flex sensors are unsuitable for open-type gloves. Moreover, IMU sensors can detect hand movements and allow for a greater degree of freedom than flex sensors.

Apart from obtaining finger motion sensor data, the noise in the raw data must be reduced to minimize tracking errors. The least mean square (LMS) and weighted Fourier linear combiner (WFLC) algorithms, low-pass filter (LPF), and Kalman filter are candidate noise-reduction methods. The Kalman filter can be used to reduce the motion-tracking noise of a glove for VR applications [36,37]. The Kalman filter shows good compatibility with IMU sensors in terms of accuracy and noise reduction. However, this filter requires a relatively long processing time compared to the LPF [38] because of its structural characteristics that necessitate recursive use of the prediction and update stages. Weill–Duflos et al. compared different advanced filters to reduce noise in velocity estimation of haptic feedback system [39]. The authors found out a realistic model of the Kalman filter can reduce latency. However, this method requires accurate identification of mechanical parameters, and it also adds latency in achieving the best estimation in accuracy. An LPF can reduce the processing time, albeit at the cost of accuracy. Moreover, LPFs also have a phase difference delay, which adds to the latency associated with data processing. Furthermore, the delay caused by an error in the sensing or processing part data required for correction may hamper users’ immersion.

### 1.3. Contribution of the Present Study

The problems faced in motion detection and reducing latency can be solved using the proposed rotary position sensor and the MMS filter. In this paper, we propose a low-latency haptic glove that can process data with extremely low latency and accurately track hand motions. If a haptic interaction is performed with both motion detection and tracking, which is the same as the VWD, the difference in motion or position between the hands in the RWD and the VWD and the latency must fulfill the haptic communication requirements to provide immersion. The MMS filter helps the proposed LLHOG system reduce errors due to differences in glove users’ finger/hand size in a VR system. The open-type glove eliminates the inconvenience caused by sharing gloves with other people.

Table 1 describes the features of the proposed haptic gloves and conventional haptic gloves to enable VR immersion. Conventional haptic gloves include flex sensor + LPF and IMU sensor + Kalman filter, whereas our proposed haptic glove is based on rotary position sensor + MMS filter. Conventional gloves can be open or closed, and our proposed glove is open to accommodate a finger length variant. The flex sensor-based gloves require two sensors per finger to track hand motion. Likewise, the IMU sensor-based gloves require at least one sensor per finger to track hand motions. By contrast, the proposed glove uses two small rotary position sensors per finger to track hand motions accurately. The flex sensor-based gloves cannot track adduction/abduction, whereas the IMU sensor and rotary position sensor-based gloves can accurately measure flexion/extension and adduction/abduction. Latency in terms of data processing is shorter in the case of flex sensor-based gloves. The IMU sensor-based gloves have a longer data processing latency. On the other hand, the proposed glove has the shortest data processing latency among the gloves mentioned above. The proposed glove has strong robustness to the finger length variance than the conventional gloves. In the current manuscript, we provide an overview of the architecture of the proposed LLHOG using the MMS filter. Then, we present the LLHOG for the immersive VR interaction in more detail, followed by an evaluation of its performance in terms of its accuracy, processing delay, and the effect of finger length on the performance.

The remainder of this paper is organized as follows. Section 2 presents an overview of the proposed glove, along with the proposed calibration and filtering method. In Section 3, the performance evaluation results obtained in this study are discussed, along with processing delay and hand motion accuracy. Finally, our concluding remarks are presented in Section 4.

## 2. System Overview

**Figure 2:**
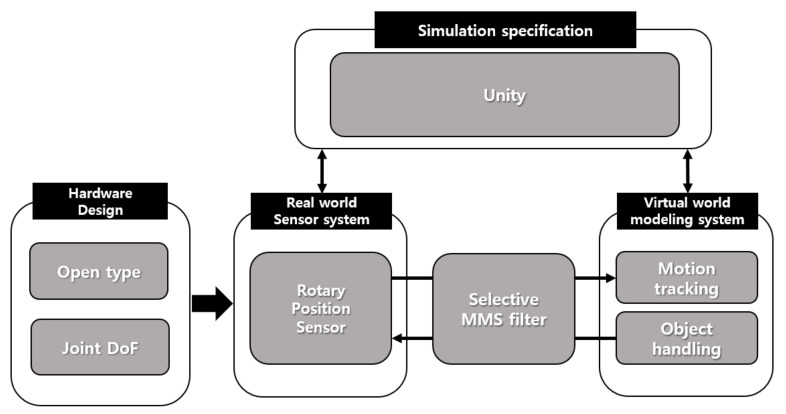
System overview of the proposed haptic glove.

Figure 2 illustrates an overview of the working principle of the proposed glove. The hardware part includes the hardware design and the sensor system. The hardware architecture of the glove is designed to fulfill three requirements. First, the glove hand surface should be open so that users can wear the glove comfortably. Second, accurate motion detection must be enabled with two position sensors per finger; therefore, it should be designed with a joint DoF structure. Third, it must be implemented in a form that can respond to the haptic feedback data received from the VWD. The sensor system of the proposed glove includes two 3382H-1-103-rotary position sensors placed in MCP joints. It detects finger motion and represents the magnitude of finger motion in terms of a resistance value. Both sensors are connected to the Arduino Nano 33 BLE development board. The MMS filter is implemented on the Arduino development board to scale the obtained sensor value between 0 to 1 for reducing noise and increasing the motion-tracking precision. The MMS filters increase the accuracy of motion-tracking in the VWD. The processed signal is sent to Unity software via BLE communication to create an immersive experience, where users can interact with and manipulate virtual objects.

### 2.1. Rotary Position Sensor

**Figure 3:**
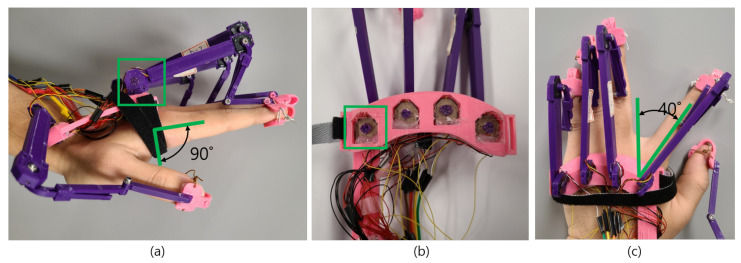
Proposed haptic glove with rotary position sensor placed in MCP joints. (**a**) rotary position sensor placed in the upper structure of the glove connected with exoskeleton hand to measure flexion/extension. (**b**) another rotary position sensor placed in the lower structure of the proposed glove connected to the exoskeleton hand with knob to measure adduction/abduction. (**c**) demonstration of performing adduction and abduction ranging from $0^{\circ }$ to $40^{\circ }$.

A rotary position sensor measures the displacement of any object and represents the displacement as electrical signals. The working principle of a rotary position sensor is identical to that of a potentiometer. The position sensor contains a section composed of carbon through which electricity can flow, and when a current flows, the carbon section acts as a resistor, and the sensor resistance changes as the length of the carbon section changes [40]. Park et al. used two linear potentiometer sensors to measure finger motions [41]. However, there are significant errors in measuring the motions of the fingers. Moreover, the linear potentiometer sensor is suitable for closed hand gloves and cannot be used in open or exoskeleton-based gloves. By contrast, Othman et al. demonstrated that a rotary potentiometer sensor could be used to measure finger flexion [42]. They proposed that a rotary potentiometer sensor can be placed in finger joints to measure finger displacement. Moreover, they compared the rotary sensor with a flexible bend sensor and found that the rotary potentiometer sensor yields accurate values, while the accuracy of the bend sensor decreases gradually. In the case of haptic gloves, it is important to determine finger motion extremely accurately; hence, we have used the 3382H-1-103 rotary position sensor in our proposed gloves. It is a 10-kΩ small potentiometer, and it consumes minimal amounts of power, which is essential for haptic gloves. As the resistance acts over the length of the carbon through which the current flows, the resistance increases gradually as the length of the carbon section increases. When such a sensor is located at the glove’s MCP joint, the resistance changes according to the degree of bending of the finger. Figure 3a–c show the body of the haptic glove, which measures the flexion/extension and adduction/abduction through the rotary position sensors placed in the MCP joints. Figure 3a shows one rotary position sensor is placed on the upper side of the glove structure and attached to an exoskeleton structure. The exoskeleton structure itself is attached to the fingertip, such that when a user performs flexion/extension, the inner section of the position sensor moves in tandem with the finger’s angular movement. In this study, the range of angular movements for flexion/extension is $0^{\circ }$ to $90^{\circ }$. Figure 3b shows another rotary position sensor placed in the lower part of the glove structure to measure adduction/abduction. The sensor is attached to the exoskeleton structure with a knob, such that when a user performs the adduction/abduction, the sensor’s inner section moves according to the angular movement produced by the adduction/abduction. In this study, the range of angular movements for adduction/abduction is set to $0^{\circ }$ to $40^{\circ }$, which can be seen in Figure 3c.

Using the structure shown in Figure 3, the movement can be accurately identified with only two small position sensors, as opposed to the flex-sensor-based glove structure that requires the hand to be wrapped. Differences in finger lengths across users of VR applications do not affect the wearability of the proposed glove.

**Figure 4:**
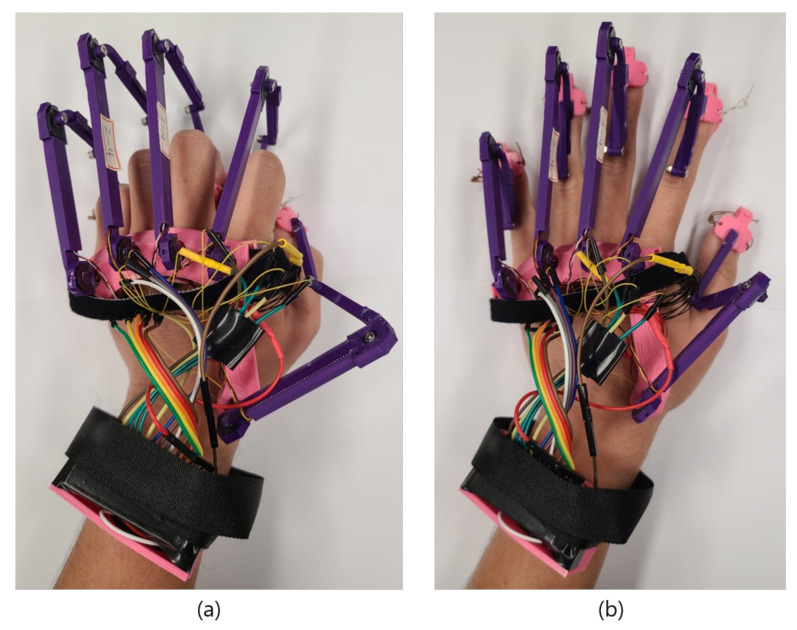
Flexion and extension movements with proposed haptic glove. (**a**) demonstration of flexion movements of $90^{\circ }$ which is considered to be the maximum flexion value in the proposed haptic glove. (**b**) demonstration of extension movements of $0^{\circ }$ which is considered to be the extension value in the proposed haptic glove.

It models the hand motion in the VWD using sensing data that varies depending on the joint position and angle that changes in the gloved hand. The accurate detection of finger motions, such as flexion, extension, adduction, and abduction, enables most finger movements to be accurately modeled. Figure 4a,b show the motions of flexion and extension, and the LLHOG senses the data necessary for modeling from the position sensors located in the MCP joints. The MCP joint is the joint where the finger and palm are connected. Humans can perform flexion up to 90°, and the angle of the active extension is 0°, which are considered the input values of the MMS filter when it is applied to the raw data of the position sensors.

**Figure 5:**
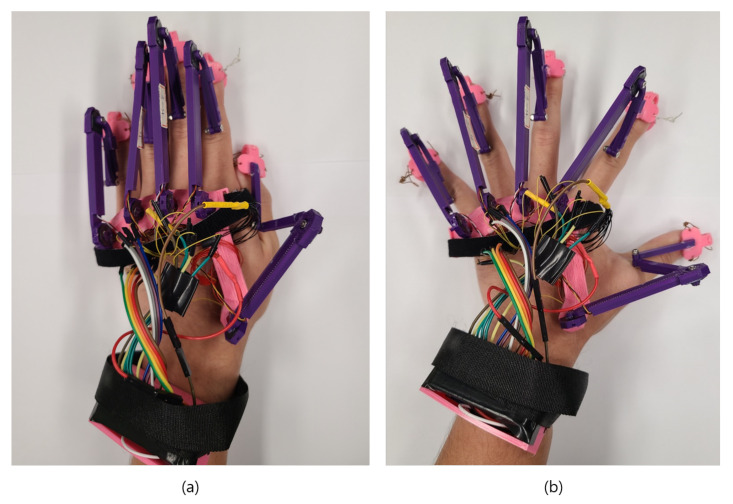
Adduction and Abduction movements with proposed haptic glove. (**a**) demonstration of adduction movements of $0^{\circ }$ which is considered to be the base adduction value in the proposed haptic glove. (**b**) demonstration of abduction movements of $40^{\circ }$ which is considered to be the maximum abduction value in the proposed haptic glove.

Figure 5a,b show the adduction and abduction motions required for modeling. The sensor data required for modeling these motions were measured from another position sensors located at the MCP joints. The range of adduction motion is considered from 0° to a maximum of 40° [43] in terms of angular displacement.

### 2.2. MMS Filter

The MMS filter scales and translates each signal in a range of 0 to 1. Moreover, the MMS filter is employed to normalize datasets in machine learning. The raw data obtained from the position sensor contain noise. If these data are used to perform modeling in the VWD without post-processing, the model accuracy will decrease. The two most widely used noise-reduction filters are the low-pass filter (LPF) and the Kalman filter. The LPF is mostly used for flex sensors, and the Kalman filter is used for IMU sensors. Each filter has advantages and disadvantages, and the flex sensor data through the LPF are subject to an additional calibration process according to the size of the user’s finger/hand, which reduces the accuracy compared to that when using a Kalman filter. By contrast, the processing of IMU sensor data with the Kalman filter easily guarantees accuracy. However, this method is unsuitable for real-time interaction due to the processing time required. The MMS filter provides a method for equally correcting a modeling result that varies according to each user’s finger length. For example, scaling is performed to provide a constant output value of the position sensor when the joint’s degree of bending is the same for different finger lengths. The formula of the min-max filter that enables scaling is as follows:

(1) $$Scaling\;value=\frac{X_{t}-min}{max-min}$$

The max and min values of the MMS filter correspond to the flexion/extension and adduction/abduction values. The flexion value of $90^{\circ }$ output by the sensor is considered the min value of the MMS filter, and the extension value of $0^{\circ }$ is considered the max value of the MMS filter. As for adduction/abduction, $0^{\circ }$ adduction is considered the min value of the MMS filter, and $40^{\circ }$ abduction is considered the max value of the MMS filter. Current sensor readings are stored to $X_{t}$, and the scaling value is calculated with the same result in the range of 0 to 1 for the same flexion angle for any user’s sensor data.

### 2.3. Calibration with MMS Filter

Two rotary position sensors were placed in the MCP joints to detect finger flexion/extension and adduction/abduction movements. The user was asked to do flexion and extension movement for five seconds to acquire value maximum and minimum value of the position sensor placed on each finger’s MCP joints. The maximum value was used as the flexion value, and the minimum value was used as the extension value for each finger. The same process was repeated to acquire adduction/abduction values. The user was asked to do abduction and adduction movements for five seconds to acquire the position sensor’s maximum and minimum value placed on each finger’s MCP joints. The MMS filter was applied to the acquired data to scale the values from 0 to 1 to accommodate finger length variance. The proposed Algorithm 1 for calibration with the MMS filter uses the flexion/extension sensor data $F_{raw}$ and adduction/abduction sensor data $A_{raw}$ as the input. The output includes flexion/extension filtered data $F_{fil}$, and adduction/abduction filtered data $A_{fil}$.

We first initialize sensor variables and assigned analogRead to the variable. Then we set $F_{min}$, $A_{min}$ value to 9999 and $F_{max}$, $A_{max}$ to 0 for calibration purposes. Calibration time for flexion/extension $F_{thres}$ and adduction/abduction $A_{thres}$ is set 5 s and 10 s, respectively. After powering up the haptic gloves, the first 5-s window is used as the calibration time for flexion and extension. After that, another 5-s window is used as the calibration time for abduction and adduction. The millies() function is used to facilitate the calibration as the millies() function calculates the times in ms after powering up the glove. The millies() function facilitates the division of the calibration time into 5-s windows. The calibration of flexion and extension starts by comparing the millies() and $F_{thres}$. While the millies() is less than $F_{thres}$; If the $F_{raw}$ is greater than $F_{max}$, the system assigns the value of $F_{raw}$ to $F_{max}$, which will be used as the extension value. If the $F_{raw}$ is less than $F_{min}$, the system assigns the value of $F_{raw}$ to $F_{min}$, which will be used as the flexion value. While the millies() is less than $A_{thres}$; If the $A_{raw}$ is greater than $A_{max}$, the system assigns the value of $A_{raw}$ to $A_{max}$, which will be used as the adduction value. If the $A_{raw}$ is less than $A_{min}$, the system assigns the value of $A_{raw}$ to $A_{min}$, which will be used as the abduction value. After calibration, the system implements the MMS filter by minimizing $F_{raw}$ to $F_{min}$ and dividing them by minimizing $F_{max}$ to $F_{min}$ for flexion/extension. The same procedure is also used for adduction/abduction. However, the output of the MMS filter is a floating value between 0 and 1, which adds to the complexity of modeling hand motions in Unity and causes delay. Therefore, we converted the floating points to an integer value to better represent data and reduce the processing delay.
**Algorithm 1:**Proposed calibration method with the MMS filter.
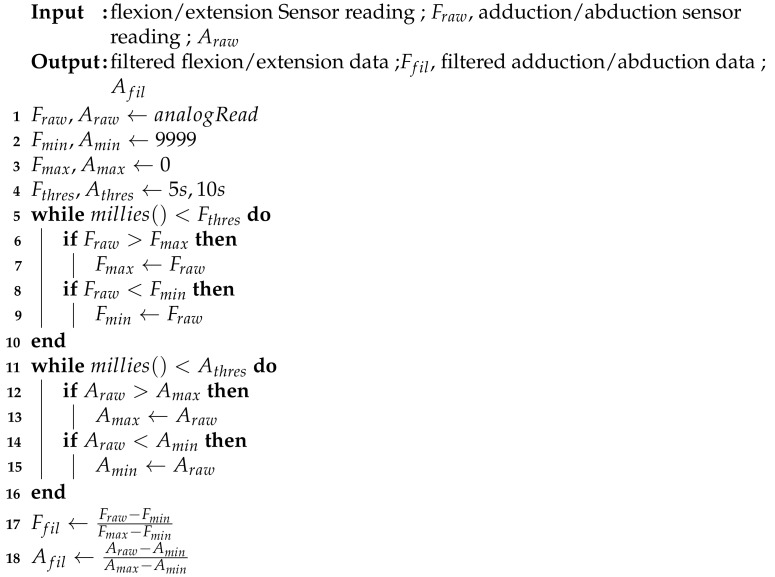


**Figure 6:**
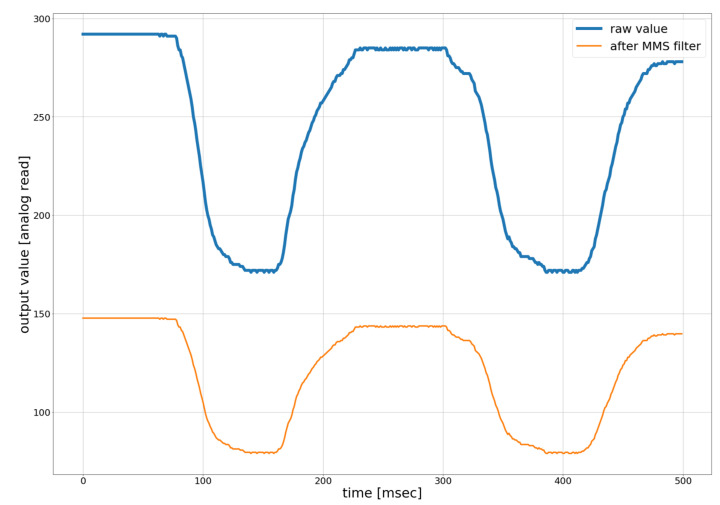
Raw and filtered values of the proposed calibration method with the MMS filter. The x-axis represents the time in microseconds and y-axis represent the analog output value from sensors before and after applying MMS filter.

Figure 6 shows the raw values of the position sensor and the filtered sensor value. As the filtered value ranges from 0 to 1, it is not easy to plot both of them in the same graph. To facilitate the plotting filtered sensor value, it was multiplied by 100 and was added 50 for each sensor reading. It can be seen that the output value of the MMS filter has minimal processing delay and looks stable. The raw value of the rotary position sensor has low noise due to good resolution. The MMS filter is used to scale and typecast floating-point values to integer values to ensure fast data processing for VR in Unity and low processing/communication delay. We have observed that the filtered sensor value is more stable than the raw data for facilitating motion-tracking in VR.

### 2.4. Object Handling CONTROL Data

The glove presented in this paper uses an interface that enables interaction between the RWD and the VWD. It is essential for any virtual reality environment to accurately track real-time hand motions so that the same motions can be reproduced in VWD. We have used Unity software to resembles the RWD hand motion in VWD. We transferred the real-time hand motion data processed with the MMS filter to Unity to reproduce virtual hand motions.

**Figure 7:**
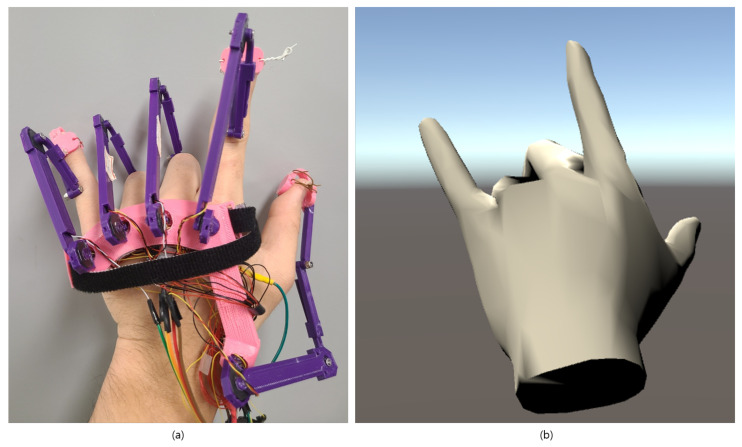
(**a**) Motion in real-world domain; (**b**) modeling result in the virtual-world domain which was done in Unity Software.

Figure 7a,b show hand motions in the real-world domain and the virtual-world domains, respectively. The user was asked to perform horn symbol motion RWD, which can be seen in Figure 7a, and real-time hand motion was reproduced in Unity virtual environment, which can be seen in Figure 7b. Figure 7 demonstrates that accurate hand motion can resemble in VWD with our proposed glove.

## 3. Performance Evaluation

Performance evaluation of the proposed haptic gloves was divided into two parts: processing time and accuracy from the perspective of different hand sizes. Brief descriptions of these parts are as follows:

### 3.1. Processing Time

**Figure 8:**
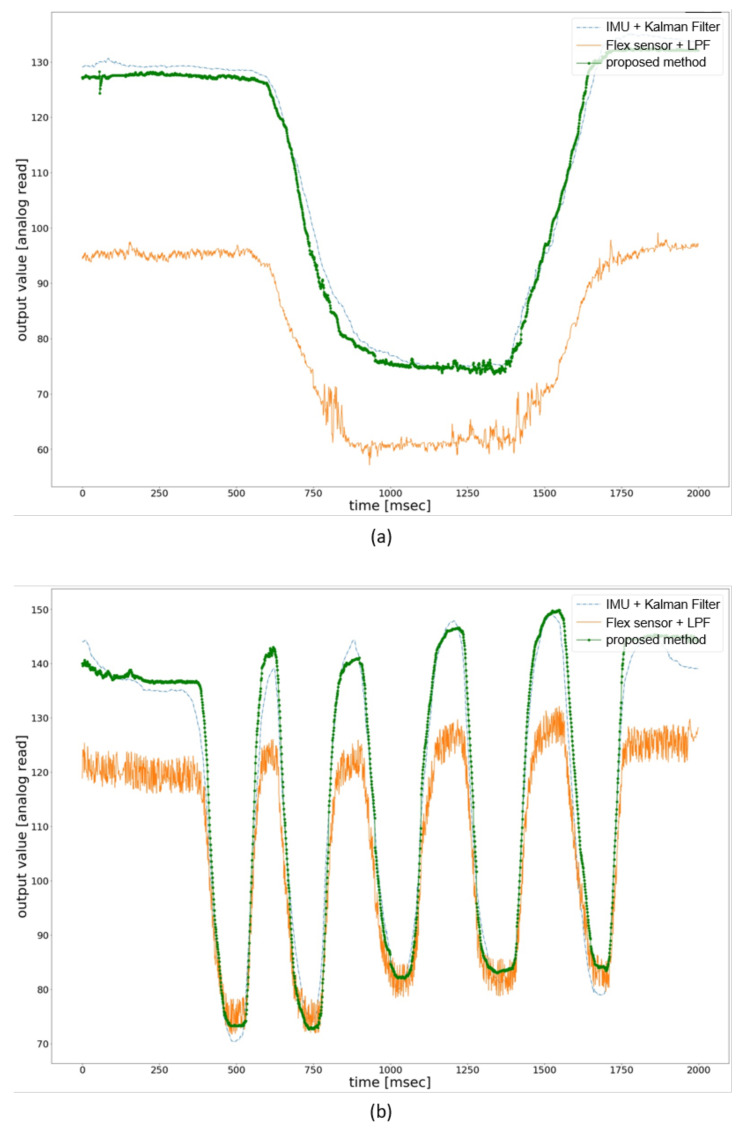
Comparison of the filtered flexion/extension value in terms of noise reduction with method 1(IMU sensor + Kalman filter) and method 2(flex sensor + LPF). (**a**) noise-reduction comparison for normal flexion/extension movements. (**b**) noise-reduction comparison for fast flexion/extension movements.

Figure 8 compares the existing methods’ filtered output value with our proposed method for flexion and extension. The x-axis represents the time in microseconds, and the y-axis represents the filtered value of the analog reading from the sensor. The filtered value was multiplied by 100 and was added 50 for each sensor reading to facilitate the plotting. We have used IMU, flex sensors for the evaluation process because these sensors are frequently used in the haptic glove for hand motion-tracking in VR. The method used in this paper includes IMU sensors + Kalman filter filtered value and flex sensors + LPF filtered value. We have used the Kalman filter library from Arduino documentation was used for Kalman filter-based method. Process noise variance for the accelerometer and the gyro bias was set to 0.001 and 0.003, respectively. Moreover, measurement noise variance was set to 0.03. As for LPF-based method, we have used the first order LPF and have used 4 Hz as the cutoff frequency. Data processing with MMS filter was done on an Arduino Nano 33 BLE microcontroller board and transferred to python 3.6 scripts through pyserial library to plot the processed data. Figure 8a shows the filtered value of the three methods used to evaluate performance. The user was asked to perform flexion and extension within 2000 milliseconds, and the raw value was processed and plotted against the time. It can be seen that method 1 and the proposed method have similar filtered values with little noise than method 2. Moreover, the user was also asked to perform the flexion and extension five times within 2000 milliseconds. Figure 8b shows the filtered value of fast flexion and extension with the same three methods. The proposed method works better in the fast movement, and the noise level is less than the other two methods.

**Figure 9:**
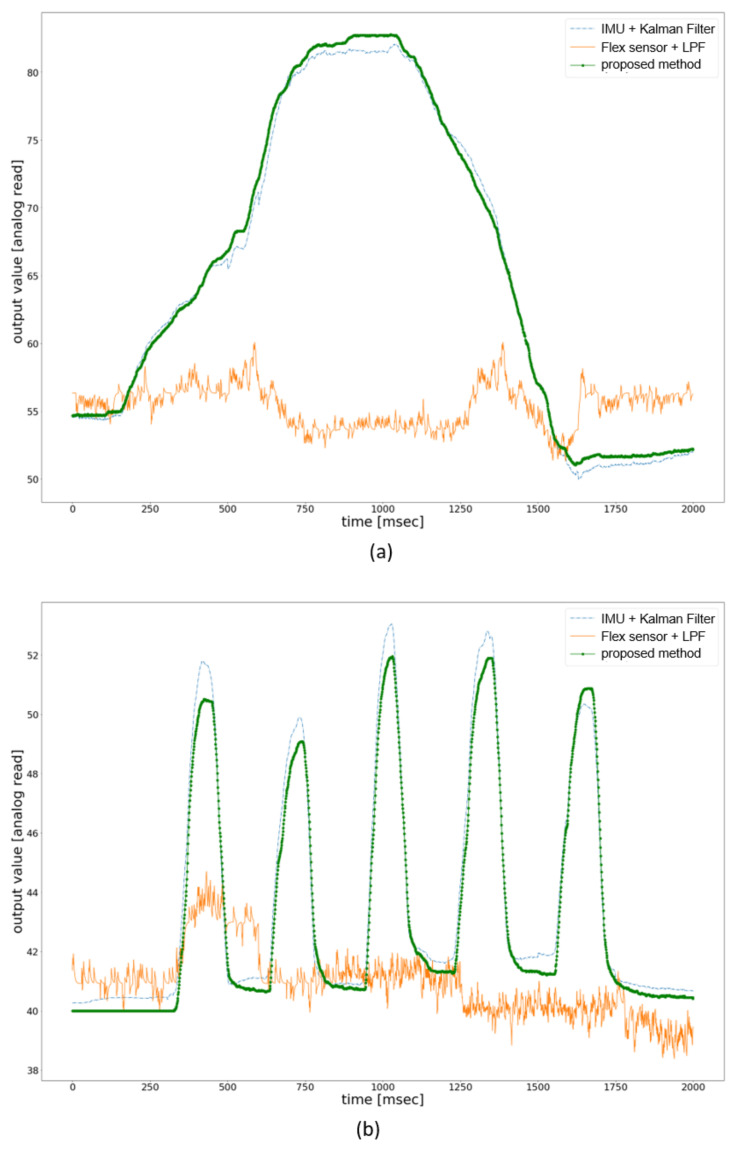
Comparison of the filtered adduction/abduction value in terms of noise reduction with method 1(IMU sensor + Kalman filter) and method 2(flex sensor + LPF). (**a**) noise-reduction comparison for normal adduction/abduction movements. (**b**) noise-reduction comparison for fast adduction/abduction movements.

Figure 9 compares the existing methods’ filtered output value with our proposed method for adduction and abduction. The experimental setup was the same as for flexion/extension measurement in Figure 9. The x-axis represents the time in microseconds, and the y-axis represents the filtered value of the analog reading from the sensor. The MMS filter’s output value is in the range of 0 to 1, so the filtered value was multiplied by 100 and was added 50 for each sensor reading to facilitate the plotting. Figure 9a shows the filtered value of the three methods used to evaluate performance. The user was asked to perform adduction and abduction within 2000 milliseconds, and the raw value was processed and plotted against the time. It can be seen that method 1 and the proposed method have similar filtered values with little noise, whereas the filtered value of method 2 produces greater noise, which is not suitable for a virtual environment. The user was also asked to perform the adduction and abduction five times within 2000 milliseconds. Figure 9b shows the filtered value of fast adduction/abduction with the same three methods. The proposed method works better in the fast movement, and the noise level is less than the other two methods. However, it should be noted that the MMS filter is not a traditional noise cancelling filter. It scales the data from 0 to 1 and presents the data in a floating-point number. In the proposed glove, we have converted the floating-point output value to the integer value to enable data to use in VR and achieve low latency. The use of MMS filter with sensors with significant noise can affect the accuracy in motion-tracking.

Latency is significant for a haptic glove, which is used in the virtual environment to manipulate objects. In some environments, the latency requirement is as low as 1 ms, so making haptic gloves with less processing delay for virtual interaction is crucial. Table 2 lists the average processing delay of the IMU sensor, flex sensor, and rotary position sensor data processed with the LPF, Kalman filter, and MMS filter for index finger. All measurements were conducted 100 times, and the average processing time was calculated with 100 sensor readings. For the IMU sensor, we only used the Kalman filter because it is hard to derive the standard output value with LPF and MMS filter, owing to the gyro drift phenomenon that occurs when processing the raw data of the 9-DoF IMU sensor that uses the gyro sensing value. We observed that IMU sensor data processed with the Kalman filter have an average processing delay of 1920.36 μs, which is very high. The reason behind the high processing delay is the complexity of the calculation algorithm for processing and predicting values. The flex sensor data processed with the Kalman filter, LPF, and MMS filter have a processing delay of 738.02 μs, 280.02 μs, and 145.64 μs. Here, we can also see that data processing of flex sensor with Kalman filter takes longer than other filters. LPF takes considerably less time than Kalman filter to process data because of the algorithm’s simplicity. We can see that the flex sensor processed with the MMS filter has less processing delay than the other two filters because the MMS filter scales the data from 0 to 1 in floating points and converts the data into the integer. One potential reason could be requiring a lower complexity algorithm in scaling and cutting off floating points. We have also implemented the Kalman filter, LPF, and the MMS filter on our proposed glove, and the processing delays are 370.95 μs, 280.69 μs, and 145.37 μs, respectively. We have observed a similar processing delay for the flex sensor and rotary position sensor with the MMS filter because they share the same sensing principle.

### 3.2. Finger Motion-Tracking ACCURACY

Finger motion-tracking-based haptic gloves are crucial in almost every application involving immersive interaction, rehabilitation, and teleoperation [44,45,46]. Researchers are trying to develop robust haptic gloves with very high accuracy in finger and hand motion-tracking. Lu et al. developed a 3-D finger measurement system with strain sensors placed in interphalangeal (IP), MCP, and carpometacarpal (CMC) joints to measure finger movement accuracy [47]. They measured the estimated error for flexion-extension and adduction-abduction movement and observed a mean error of less than $3.5^{\circ }$ across all movements. Li et al. presented a hand motion measurement system with 14 custom-made bending sensors and an IMU sensor [48]. Experiments were conducted on six subjects with different hand sizes, and they were asked to execute nine grasping motions. The mean absolute error (MAE) of the proposed system was $6.35^{\circ }$±$0.92^{\circ }$. Jun et al. also propose a wearable real-time hand measurement algorithm for different hand sizes. They used fiber Bragg grating (FBG) strain sensors and 3D printed hand replica with different hand sizes to measure hand motions [49]. They Measured the angle error for DIP, PIP, and MCP joints and observed a mean error angle of $0.47^{\circ }$±$2.51^{\circ }$ and a MAE of $1.63^{\circ }$±$1.97^{\circ }$. Gajdosik and Bohannon stated that the allowable mean error of finger movements should be less than $5^{\circ }$ [50]. This paper uses $5^{\circ }$ as a standard error value and compares it with our proposed haptic gloves motion error in terms of flexion/extension and adduction/abduction. In this paper, we measured the hand motion for index fingers of ten participants and compared the MAE with the finger’s actual angular movements.

**Figure 10:**
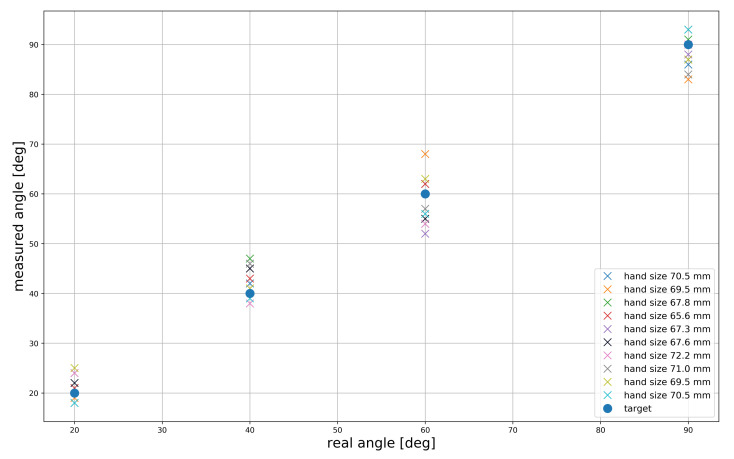
Mean error for flexion and extension in terms of real angle and measured angle.

Ten healthy participants with different hand sizes were selected to measure the hand motion accuracy of the proposed glove. Finger sizes were measured from the MCP joints to the fingertips using a scale, and the finger sizes were 68.9 ± 3.3 mm. In this paper, we evaluate the mean error for index fingers of all participants. First, the participants were asked to wear and calibrate the gloves for 10 s. Then, they were asked to perform flexion and extension movements over the range of $0^{\circ }$ to $90^{\circ }$. After flexion and extension, participants were asked to move their finger to $20^{\circ }$, $40^{\circ }$, $60^{\circ }$, and $90^{\circ }$ to measure the angle and compare it with the real angle. Then, we calculated the mean error with respect to the real angle. Figure 10 shows the mean error of the different finger length sizes in terms of flexion and extension. For $20^{\circ }$ movements, the participant with the index finger size of 69.5 mm had a large error of $0.68^{\circ }$ + $4.32^{\circ }$. For $40^{\circ }$ movements, the participant with the index finger size 67.8 mm had a large error of $0.38^{\circ }$ + $6.5^{\circ }$. However, the mean error was very high when participants were asked to move their fingers to $60^{\circ }$. Participants with hand sizes of 69.5 mm and 67.3 mm had the greatest mean errors of $0.58^{\circ }$ + $7.36^{\circ }$. Lastly, the participant with the index finger size of 69.5 mm had a large mean error of $0.38^{\circ }$ + $6.5^{\circ }$ for $90^{\circ }$ movement.

**Figure 11:**
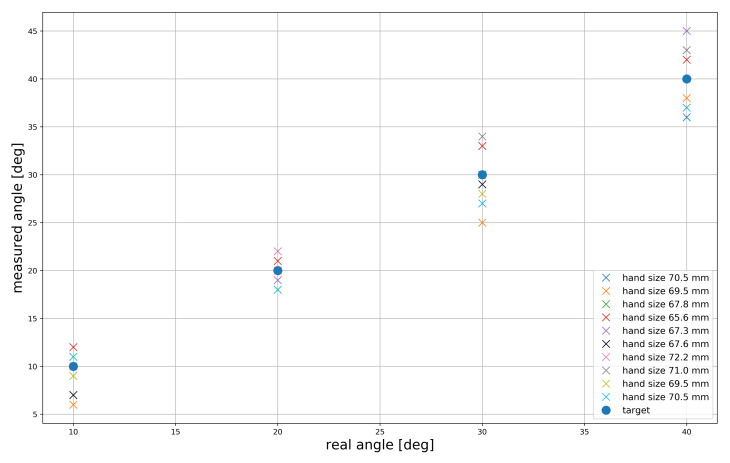
Mean error for adduction and abduction in terms of real angle and measured angle.

Figure 11 shows the mean error for the different finger lengths for adduction and abduction. The participants were asked to perform adduction and abduction over the range of $0^{\circ }$ to $40^{\circ }$. Then participants were asked to perform abduction to $10^{\circ }$, and the largest mean error was observed $0.38^{\circ }–3.62^{\circ }$ in the case of the participant with the finger length of 69.5 mm. At $20^{\circ }$, the mean errors were low for all the participants, and the largest mean error was $0.50^{\circ }$+$1.50^{\circ }$. However, we observed that the largest mean error among all the participants occurred when they performed abduction to $30^{\circ }$. The largest mean error observed was $0.50^{\circ }–4.50^{\circ }$, in the case of the participant with a finger length of 69.5 mm. Finally, the largest mean error in the case of $40^{\circ }$ abduction was $0.68^{\circ }$ + $4.32^{\circ }$ for the participant with a finger length of 67.3 mm.

Table 3 shows the individual MAE for all participants in terms of flexion-extension and adduction-abduction. The largest MAE for flexion and extension was $4.50^{\circ }$, and the participant who contributes to the error had a finger length of 67.8 mm. The least MAE for flexion and extension was $2.25^{\circ }$, and the participants who contributed this error had finger lengths of 70.5 mm and 65.6 mm. By contrast, the largest MAE for all the participants in terms of adduction and abduction was $3.00^{\circ }$, and the participant who contributes to this error had a finger length size of 67.3 mm. The least MAE for adduction and abduction was $1.75^{\circ }$, and the participant who contributed this error had a finger length of 70.5 mm. We calculated the average MAE for flexion-extension and adduction-abduction as $3.091^{\circ }$ and $2.068^{\circ }$, respectively.

Table 4 shows the comparison of different glove motion-tracking errors with the proposed glove. Lu et al., in their paper, used strain sensors to track finger motion, and the authors observed a mean error of $3.5^{\circ }$ for all movements [47]. On the other hand, Jun et al. used the FBG strain sensor to track hand motion, and they observed a mean error of $1.63^{\circ }$±$1.97^{\circ }$[49]. Li et al. have used the IMU sensor to track hand motion; however, they observed a more significant mean error than other gloves, which is $6.35^{\circ }$±$0.92^{\circ }$[48]. Gu et al., in their glove, have custom rotational sensors and observed a mean error of $0.5^{\circ }$ in hand motion-tracking, which is very promising and has higher accuracy than other methods [29]. BeBop developed fabric bend sensor-based data gloves to track hand motion for commercial use, and they claim that they have observed ±$1.5^{\circ }$ error in tracking motion [51]. Our proposed gloves use rotary position sensors to track hand motion, and we have observed a MAE of $3.091^{\circ }$ for flexion-extension and $2.068^{\circ }$ for adduction-abduction. It can be seen that the motion-tracking with rotary position sensor achieved considerable accuracy along with other methods. However, this error can be minimized by placing a sensor in the DIP and PIP joints to track finite fraction joint displacement through a rotary position sensor. Although the glove proposed by Gu et al. has a very low motion-tracking error of $0.5^{\circ }$, the allowable motion-tracking accuracy error specified by Gajdosik and Bohannon is $5^{\circ }$. Our proposed glove has achieved a motion-tracking error of $3.091^{\circ }$ and $2.068^{\circ }$, which is under the $5^{\circ }$ threshold. The motion-tracking accuracy of the proposed glove can be improved using more rotational sensors in the PIP and DIP joints of the finger. Moreover, the rotational sensors for flexion-extension and adduction-abduction movement are placed on the upper structure of the glove, and the structure has empty spaces between the joint and sensors. In the future, we plan to shorten the gap between exoskeleton and rotary axis hole of sensor to measure the precise movement of flexion-extension and adduction-abduction.

Table 5 shows the comparison of latency of different VR gloves with the proposed glove. Lu et al., in their paper, used strain sensors to track finger motion, but the author did not calculate the latency of data sensing and motion-tracking [47]. Li et al. have used the IMU sensor and bend sensor, and they have observed a response time of 24.35 ± 1.54 ms for bending sensor output [48]. Jun et al. used the FBG strain sensor to track hand motion, and they found out that it takes 20–40 ms to track the finger joint [49]. Gu et al. have used custom rotational sensors to measure the finger’s bending and observed delay 20–40 ms, including data acquisition, data processing, and force-feedback unit system [29]. BeBop data gloves have used a fabric bend sensor to track finger movement and achieved a response time of 6 ms for tracking finger movement [51]. In our proposed gloves, we have used rotary position sensors to track finger motion, and we have achieved a very low latency of 145.37 μs, which includes data acquisition and processing delay for one finger. We have also calculated the data processing latency for the whole hand, which is less than 1 ms. In the experiment, BLE communication is used to transfer the data from Arduino to PC to resemble hand motions in the VR environment. A latency of 4 ms in tracking hand motion in VR has been observed which includes data acquisition, data processing, data transmission and motion-tracking in Unity. However, the latency can vary in the case of using WiFi or 5G infrastructure, which can be one millisecond to tens of milliseconds. Although the proposed glove’s motion-tracking accuracy is a little lower than some of gloves mentioned in Table 4, our proposed glove has a very low latency of 4 ms, which is suitable for seamless VR immersion.

## 4. Conclusions

Immersive VR applications require that immersion be guaranteed through fast interaction between the VWD and the RWD through wearable peripherals. To this end, we proposed a low-latency haptic open glove (LLHOG), which enables real-time interaction between the RWD and the VWD through a wearable five-finger glove. The LLHOG senses hand and finger motions using rotary position sensors placed in the MCP joints. The position sensors and the MMS filter of the LLHOG are used to achieve low latency, high accuracy, and robustness to finger length. The proposed glove achieved 145.37 μs processing delay per finger and 4 ms hand motion-tracking delay for whole hand, which is superior to other methods. The average MAE for flexion and extension was $3.091^{\circ }$, and the average MAE for adduction and abduction was $2.068^{\circ }$, which means that the LLHOG is suitable for use in any immersive interaction. Our results confirm that the LLHOG is robust to variations in finger length. This study is done to achieve low processing delay and higher hand motion-tracking accuracy. Although the experiments suggest that the proposed glove is suitable for motion-tracking in VR interaction, the MMS filter can perform well when a sensor has very low noise. Using the proposed MMS filter which is not a pure noise filter with sensors with significant noise may affect the motion-tracking accuracy. The MCP joints displacement angle of the proposed glove from 0° to 90° may be disputable as some researchers suggested that it can be from 0° to 100°. In addition, to manipulate virtual objects in VR, the hand’s absolute position in the space is crucial. In the future, we will incorporate the IMU sensor and professional controller for VR (HMDs) so that the absolute position can be calculated in the space for virtual object manipulation. Moreover, a haptic feedback system can be implemented along with the proposed gloves for improved immersive interaction.

## Figures and Tables

**Table 1:** Features of the conventional method and the proposed Haptic glove.

	Conventional Method		Proposed Method
	**Flex Sensor + LPF**	**9-DoF IMU Sensor + Kalman Filter**	**Rotary Position Sensor + MMS Filter**
Glove type	Closed	Open or Closed	Open
Number of sensors required	2 EA/finger	1 EA/finger	2 EA/finger
Motion detection accuracy	Neither adduction nor abduction detection	Accurate	Accurate
Latency	Shorter	Longer	Shortest
Robustness to finger length variance	Weak	Weak	Strong

**Table 2:** Comparison of processing delay of the proposed glove with conventional method.

	Kalman Filter	Low-Pass Filter	MMS Filter
IMU sensor	1920.36 μs	*N/A*	*N/A*
Flex sensor	738.02 μs	280.02 μs	145.64 μs
Rotary Position sensor	370.95 μs	280.69 μs	145.37 μs

**Table 3:** MAE of flexion/extension and adduction/abduction for different hand sizes.

**Finger Length**	70.5	69.5	67.8	65.6	67.3	67.6	72.2	71.0	69.5	70.5	MAE
**Flexion and Extension**	2.25	4.25	4.50	2.25	4.25	3.75	3.00	4.25	3.00	2.50	3.091
**Adduction and Abduction**	1.75	2.75	2.25	2.00	3.00	2.25	2.00	2.50	2.00	2.25	2.068

**Table 4:** Comparison of motion-tracking error with other methods found in the literature.

	Sensors	Angle Error
Lu et al. [47]	strain sensor	<3.5${}^{\circ }$
Li et al. [48]	IMU sensor + bend sensor	$6.35^{\circ }$ ± $0.92^{\circ }$
Jun et al. [49]	FBG strain sensor	$1.63^{\circ }$ ± $1.97^{\circ }$
Gu et al. [29]	rotational sensor	$0.5^{\circ }$
BeBop [51]	fabric bend sensor	± $1.5^{\circ }$
Proposed glove	rotary position sensor	$3.091^{\circ }$ and $2.068^{\circ }$

**Table 5:** Comparison of Latency with other methods found in the literature.

	Sensors	Latency
Lu et al.[47]	strain sensor	N/A
Li et al. [48]	IMU sensor + bend sensor	24.35 ± 1.54 ms
Jun et al. [49]	FBG strain sensor	20–40 ms
Gu et al. [29]	rotational sensor	20–40 ms
BeBop [51]	fabric bend sensor	6 ms
Proposed glove	rotary position sensor	4 ms

## Data Availability

Not applicable.
